# Psychological Diagnoses and Weight Loss among Appalachian Bariatric Surgery Patients

**DOI:** 10.1155/2020/1743687

**Published:** 2020-02-10

**Authors:** Makenzie L. Barr, Cassie Brode, Lawrence E. Tabone, Stephanie J. Cox, Melissa D. Olfert

**Affiliations:** ^1^West Virginia University, Davis College of Agriculture, Natural Resources and Design, Division of Animal and Nutritional Sciences, Department of Human Nutrition and Food, Morgantown, WV, USA; ^2^Department of Behavioral Medicine and Psychiatry, West Virginia University, School of Medicine, Morgantown, WV, USA; ^3^Department of Surgery, West Virginia University, School of Medicine, Morgantown, WV, USA

## Abstract

**Background:**

The relationship between presurgical psychopathology and weight loss following bariatric surgery is complex; previous research has yielded mixed results. The current study investigates the relationship among presurgical mental health diagnoses, symptom severity, and weight loss outcomes in an Appalachian population, where obesity-related comorbidities are prominent.

**Methods:**

A retrospective chart review was performed on bariatric surgery patients in an accredited Appalachian centered academic hospital in northern West Virginia between 2013 and 2015 (*n* = 347). Data extraction included basic demographics, anthropometrics (percent excess weight loss (%EWL)) at six-month, one-year, and two-year postoperative visits, and two validated psychological questionnaires (Beck Depression Inventory (BDI-II) and Beck Anxiety Inventory (BAI)) from patient's presurgical psychological evaluation.

**Results:**

Average patient population was 92.5% Caucasian, 81.5% female, 45 ± 11.5 years old, and 84.1% who underwent laparoscopic Roux-en-Y gastric bypass surgery with the remaining having laparoscopic sleeve gastrectomy. At baseline, no differences were detected in weight, excess body weight, or body mass index between surgery types. Average baseline BDI-II score was 10.1 ± 8.68 (range 0–41) and BAI score was 6.1 ± 6.7 (range 0–36), and this was not significantly different by surgery at baseline. Both baseline psychological scores were in the “minimal” severity range. BDI-II was positively related to BMI of patients at baseline (*p* = 0.01). Both BDI-II and BAI were not significantly related to %EWL across follow-up.

**Conclusion:**

Other than baseline weight, BDI-II and BAI scores were not related to %EWL outcomes in patients receiving bariatric surgery in the Appalachian region. Future work should examine mixed methods approaches to capture prospective and longitudinal data to more thoroughly delve into mental health aspects of our Appalachian patients and improve efforts to recapture postoperative patients who may have been lost to follow-up.

## 1. Introduction

Obesity, a disease with adverse effects on an individual's health [1], can often extend to many other areas of a person's life including not only physical health but also quality of life, self-esteem, and psychosocial functioning [2, 3]. Individuals with obesity are at an elevated risk for developing insulin resistance and type 2 diabetes, sleep apnea, cardiovascular disease, hypertension, and increased prevalence of cancers and have higher rates of mental illnesses [4, 5]. Persons residing in the Appalachian region appear to be the most vulnerable, with rates of obesity and weight-related comorbidities (i.e., diabetes and hypertension) exceeding the national prevalence [2–7]. West Virginia, the only state entirely encompassed in the Appalachian region, houses 15.2% of adults diagnosed with type 2 diabetes mellitus compared to 12.7% of the nation (2013-2014 data) [6] and 42.7% of adults diagnosed with hypertension compared to 33.5% of the nation (2016 data) [7–9]. Along with these related comorbidities, those with excess weight tend also to have higher rates of psychological complications [10–12]. The Appalachian Regional Commission reports higher rates of mentally unhealthy days in the region compared to the nation as a whole [9]. West Virginia also leads with the highest prevalence rates of depression with 23.6% of adults diagnosed compared to 17.8% adults in the nation as a whole [7, 13]. With this link to excess weight, clinically diagnosed depression [14–16] is an essential factor to consider when treating obesity holistically.

As an approach to lessen the impact of severe obesity and its related comorbidities, bariatric surgery has been deemed to be one of the most effective treatments. Given the high rates of comorbid psychopathology and medical complexity in patients seeking bariatric surgery, severe obesity requires a multidisciplinary approach [17]. Thus, in preparation for surgery, the American Society for Metabolic and Bariatric Surgeries (ASMBS) recommends a presurgical psychological evaluation of candidates to identify psychosocial factors that may be associated with poor outcomes and to ensure proper recommendations that are made to optimize postsurgical outcomes [17–20]. This monitoring is vital, as previous literature has shown that there is a 23% prevalence rate of any mood disorder in bariatric surgery populations when compared to the United States prevalence of 10% [21]. Among depression and anxiety prevalence specifically, 19% and 12%, respectively, of bariatric surgery patients, displayed these conditions [21]. This relationship between psychological disorders, such as anxiety or depression, and weight is often bidirectional. In some studies, preexisting mental health conditions presurgically often correlate to lesser weight loss after bariatric surgery [21, 22]. However, previous research has indicated that the severity of symptoms plays a more significant role in influencing weight loss outcomes than does the presence of a mental health diagnosis overall [23].

When examining outcomes of this underrepresented population, obesity and related comorbidities are at a higher prevalence in the Appalachian region but, interestingly, Appalachian bariatric surgery is vastly understudied. The purpose of the current study was to examine surgical outcomes of an Appalachian bariatric surgery patient population based on presurgical demographics and psychosocial factors via validated psychological questionnaires.

## 2. Methods

Approval to conduct research was obtained via West Virginia University Institutional Review Board (no. 1611355277) in March 2017. Data collection was completed through a retrospective chart review of bariatric surgery patients in a large academic university practice that is accredited through the American College of Surgeons, Metabolic and Bariatric Surgery Accreditation and Quality Improvement Program (MBSAQIP). A comprehensive inclusion patient identification was conducted by a patient query of patients receiving bariatric surgery between March 2013 and July 2015 who were 18 years and older, had completed all required surgical clearances (i.e., cardiovascular, pulmonology, and psychology), and received either laparoscopic Roux-en-Y gastric bypass (RYGB) or laparoscopic sleeve gastrectomy (SG). This timeframe was used to ensure all patients in the analysis had the possibility of attending a 2-year follow-up clinic appointment. All researchers involved in data collection completed the required Health Insurance Portability and Accountability Act (HIPAA) training for clearance into patient electronic medical records (EMR). Upon EMR clearance, researchers were trained on patient chart navigation and reliability of data capturing. Data collection took place March through September of 2017. Retrieval of information was found in forms of both electronically entered data and scanned PDF files of baseline patient surveys. All data were entered into a HIPAA compliant RedCap survey. A second data pass was completed on 2% of charts to ensure data reliability of 85% or higher and reduce participant and study bias. Demographics, baseline anthropometrics, and psychological testing scores were captured via EMR. Excess body weight (EBW), percent total weight loss (%TWL: [(initial weight) − (post-op weight)]/[(initial weight) − (ideal weight)]), and change in BMI (BMI: [(initial BMI) − (post-op BMI)]) were calculated.

Utilizing the standardized outcomes reporting in metabolic and bariatric surgery for analyzing our outcome variables [24], our primary outcome measure was percent excess body weight loss (%EWL), initial weight − current weight divided by initial weight − and ideal body weight (IBW) calculated at a BMI of 25 kg/m^2^ [24]. Psychological tools used included two validated, self-reported, and psychological questionnaires (Beck Depression Inventory (BDI-II) and Beck Anxiety Inventory (BAI)) from patient's presurgical evaluation. The BDI-II is a 21-item tool used to assess the severity of depressive symptoms and is valid and reliable in bariatric populations [25]. Each item is based on a scale of 4 statements increasing in severity of each symptom. The tool is scored on a scale of 0 to 63, with higher scoring indicating more severe depressive symptoms. The BAI is also a 21-item tool used to assess the severity of anxiety symptoms in patients [26]. Each item is based on a scale of 4 statements increasing in severity of each symptom. The tool is scored on a scale of 0 to 63, with higher scoring indicating more severe anxiety symptoms.

### 2.1. Statistical Analyses

All analyses were performed using SAS (SAS®, Version 9.3) [27] and JMP (JMP®, Version Pro 13) [28]. Demographic data (sex, state, race, education, marital status, and diagnosed anxiety and depression) were reported in frequencies and percentages by surgery group at baseline and analyzed by Pearson's chi-square and Fisher's exact test when cell sizes were below 5 participants. Mann–Whitney *U* test was used for assessing association between nonparametric anthropometries and surgery group (age, height, weight, %TWL, BMI, ΔBMI, EBW, %EWL, BDI-II, and BAI) at each time point.

Linear mixed models (LMM) were used to assess postoperative changes in %EWL over a period of compared intervals (6 months, 1 year, and 2 years) by psychological scores, while controlling for demographic variables. As follow-up attendance was limited at the 2-year follow-up, LMM allows for flexibility to utilize data without “kicking out” those who did not have data at each of the follow-up points (i.e., participant attended 6-month and 2-year follow-ups but not 1-year follow-up). To fit the flexibility of the means and individual variances and covariances of the data, in addition to random and fixed effects within the model, linear mixed modeling was used. Two separate models were run on %EWL over the three-time points as BDI-II and BAI are variables highly correlated to each other (*p* < 0.01). Covariate inclusion was determined a priori for any significant demographic and baseline anthropometric as well as controlling for gender, age, and race. Best fitting models were determined through the smallest Akaike Information Criterion (AIC) and other goodness of fit indices. Null Model Likelihood Ration chi-squared test (LRT) determined model fit of covariance structure. Time was utilized as a continuous variable in both models. Best fitting models utilized autoregressive (AR1) covariance structure, correcting for degrees of freedom using the Kenward-Roger method and REML estimation, with time as a repeated variable and participant ID as the subject. For both models, post-hoc Tukey adjustments were performed for significant adjusted mean differences by group. Missing data were assumed to be missing at random and pairwise deletion was applied through LMM.

## 3. Results

In total, data were captured on patient charts who had received surgery and had the possibility of attending a two-year follow-up after surgery at the time of data collection. The sample size was based on time and data limitations leaving a sample of *n* = 347 that had complete data on sex and surgery and was used for analysis (Table 1). Data were examined for variable-specific outliers greater than 3 standard deviations above the mean, which were removed prior to analyses (*n* = 12 outliers). The bariatric surgery patients were predominately 92.5% Caucasian, 81.5% female, 64.5% married, 45 ± 11.5 years old, and 84.1% receiving RYGB surgery with the remaining receiving SG (Table 1). When stratifying the population by surgery type, similar demographic breakdowns were seen. No significant demographic differences were found between patients undergoing RYGB versus SG via chi-square analysis. Additionally, no significant differences were detected among anthropometrics by surgery group via Mann–Whitney *U* test, except for height, with patients receiving SG being slightly taller in centimeters (*p* < 0.05) which may be due to the larger percentage of the SG patients being male. Of the full cohort at baseline, 166 had ICD diagnosed depression, and 106 had diagnosed anxiety disorder(s) (ICD diagnosed 85 with both depression and anxiety disorders). Between RYGB and SG, no significant difference was found between surgeries and ICD diagnosed anxiety, depression, self-reported BAI, or BDI-II at baseline (*p*′s > 0.05). At both one- and two-year follow-ups, between the two groups of SG and RYGB, significant differences were seen among weight (*p* < 0.01), BMI (*p* < 0.01), EBW (*p* < 0.01), and %EWL (*p* < 0.01).

A significant association was identified with sex and BAI at baseline (*p* < 0.01) with females displayed higher scores than males (6.87 and 4.19, respectively). On average, for the entire cohort, BDI was related to BMI at baseline (*p* = 0.01), however, not with BAI (*p* = 0.23). When examining BDI-II, BAI, and %EWL at follow-ups, no significant differences were found.

### 3.1. Beck Depression Inventory-II Model

To examine psychological scores and their relationship with % EWL across time, LMM were used and controlled for demographic variables.  Table 2 Percent EWL null model 1 (examining BDI-II; *n* = 259 participants used) LRT was significant (*χ*^2^(1) = 194.76, *p* < 0.0001). Significant type 3 of fixed effects was found for gender: *F* (1, 211) = 6.08, *p* = 0.0145, and time: *F* (1, 293) = 11.14, *p* = 0.0010. Type 3 test of fixed effects was not significant for race: *F* (1, 204) = 0.06, *p* = 0.8111, age: *F* (1, 211) = 0.78, *p* = 0.3777, surgery: *F* (1, 420) = 1.26, *p* = 0.2618, or BDI-II: *F* (1, 212) = 0.27, *p* = 0.6039. Additionally, a surgery by time interaction was detected: *F* (1, 294) = 10.88, *p* = 0.0011(Table 2).

### 3.2. Beck Anxiety Inventory Model

Percent EWL null model 2 (examining BAI; *n* = 228 participants used) LRT was significant (*χ*^2^(1) = 175.20, *p* < 0.0001). Significant type 3 tests for fixed effects, testing overall effects, were found among gender: *F* (1, 181) = 7.65, *p*=0.0063, and time: *F* (1, 232) = 14.39, *p*=0.0002. Similar to model 1, type 3 test of fixed effects was not significant for race: *F* (1, 180) = 0.78, *p*=0.3780, age: *F* (1, 181) = 0.06, *p*=0.8004, surgery: *F* (1, 348) = 2.40, *p*=0.1225, and BAI: *F* (1, 181) = 0.18, *p*=0.6733. For time and surgery interactions of model 2, a significant interaction was detected *F* (1, 232) = 9.68, *p*=0.0021.

An additional post-hoc analysis was completed to assess the effect of comorbidities on the above multivariant analyses, by including both baseline type 2 diabetes and baseline hypertension. The two variables were chosen due to their relationship to %EWL and availability of data in this retrospective review. All variables in both the parameter estimates and type 3 tests remained at their significance level with hypertension resulting in nonsignificant effects in both BDI and BAI models (0.2387 and 0.2304, respectively), while diabetes resulted in significant effects in both models (0.0011 and 0.0065, respectively). Similarly, BDI and BAI remained nonsignificant in the models.

## 4. Discussion

Among comorbid conditions, poorer mental health is more prominent in individuals of higher weight [2–4]. When targeting morbid obesity through bariatric surgery, mental health issues can be relieved or alleviated by the surgery but can also potentially interfere with amount of weight loss after surgery [2, 29–33]. Marek and colleagues have identified, at one-year follow-up appointments, patients with elevated scores on behavioral dysfunction scales of the Minnesota Multiphasic Personality Inventory-2 (MMPI-2) predicted poorer weight loss and poorer adherence to follow-up appointments [32]. Likewise, data from a five-year follow-up found that scores on the MMPI-2 were related to poorer weight loss, while additionally recognizing presurgical Binge Eating Disorder was predictive of higher BMI [33]. In a previous study by the current authors, diagnosed type 2 diabetes, hemoglobin a1c levels, and diagnosed depression were all associated with %EWL in an Appalachian population at one-year follow-up after surgery [34]. In our current study of Appalachian surgical patients, we found that baseline BDI-II related to BMI at baseline but both BDI-II and BAI did not play a role in %EWL over time. Data did show that our population had strong outcomes of an average %EWL of 57.4%, 69.6%, and 66.6% at 6-month, 1-year, and 2-year follow-ups, respectively. Additionally, at these follow-ups, average %TWL of the full population was 27.0%, 33.2%, and 32.1%. Following criteria of successful weight loss after surgery from previous literature (50% EWL or 20% TWL), our population achieved these outcomes.

To date, a number of studies find that preoperative depression and anxiety hinder weight loss after surgery [22]; however, most studies have indicated no significant or minimal relationships between presurgical psychological factors and postsurgery weight outcomes [35, 36] and this is the same in our WV sample. Interestingly, in our patient population, the average scores of BDI-II and BAI at baseline were in the “minimal” category cut-off for both scores (a BDI-II score of 0–13 is minimal; a BAI score of 0–7 is minimal). Although there is evidence of the higher prevalence of mood disorders in bariatric surgery patients [21] and and WV residents [13], a few similar examinations of psychological profiles of bariatric surgery patients indicated these minimal symptom classifications [25]. Previous studies examining screening of depressive disorders in preoperative bariatric patients indicated the socially desirable response bias as patients understand that the psychological screening is preemptive clearance prior to receiving surgery [37, 38]. Attention to social desirability prior to surgery in an Appalachian cohort should be further explored. Likewise, qualitative data, such as clinical notes or interviews, along with other patient-reported outcomes and standardized assessments could enrich the data and delve further into the strength and weight of the score for each patient.

### 4.1. Limitations

Although this is one of the first studies examining psychological disorders and their impact in an Appalachian centered bariatric population, there are limitations. Our population was predominately Caucasian, and despite being unrepresentative of the national average, this is typical for the state of West Virginia. Further investigation should be done to capture diverse Appalachian residents. Consideration does need to be taken due to the self-reported nature of the BDI-II and BAI tools when utilizing them as a symptomatic screening method due to their face validity. Likewise, this was a retrospective review of patients receiving surgery, which is excluding patients not making the journey to receive surgery whether due to personal drop-out or not meeting criteria, and thus, data are not included. Further exploration should be taken in comparing baseline mental health measures between those receiving surgery and those who do not. Likewise, collection of additional covariates may not have been available in an individual's chart and could not be used, such as hand-written baseline documents or additional time point follow-ups. When utilizing data from this population, it is shown that there was large attrition in follow-up attendance with the ability of 347 patients to reach a 2-year follow-up, though only 108 were retained (31%). From previous literature, this is far less than average for both surgeries [39] indicating that caution should be taken to generalize these results to other populations as data from those nonretained participants could change outcomes found here.

Additionally, as this was a predictive model of psychological conditions and their impact on postsurgical outcomes, further exploration of Appalachians' postsurgery quality of life and mental health is warranted. Many other studies examine presurgery health and how they relate to postsurgical outcomes; however, methods and findings are inconsistent [40]. Although there is some exploration into postsurgery contribution of weight loss, some work suggests that when examining long-term follow-up, such as after 2 years, these presurgical variables make up less of the contribution and postsurgery variables are more predictive [41].

## 5. Conclusion

In our Appalachian bariatric surgery population and models examining %EWL across two-year postbariatric surgery, self-reported tools of depression and anxiety were not associated with weight loss outcomes. Studies indicate that some bariatric patients tend to minimize their distress, thus presenting favorably as good candidates to obtain surgical clearance. Capturing qualitative interview data of psychological well-being, in low stress environment, could be advantageous. Data may benefit from a more targeted intervention before or after surgery to improve quality and unbiased data for psychological evaluations.

Future work should examine structured diagnostic interviews, qualitative data, and postsurgery follow-ups of mental health and quality of life to ascertain patients' real-time level of distress and impact. Exploration into utilization of technology-based approaches for rural Appalachian patient may assist in retention improvements. Mixed methods' data of rural patient need for a virtual platform that could assist with follow-up data via Bluetooth technology may improve understanding of postsurgery outcomes and mental health status in hard-to-reach rural populations [40].

## Figures and Tables

**Table 1:** Descriptive statistics, by surgery type, of Appalachian bariatric surgery patients between 2013 and 2015 receiving surgery in West Virginia.

Variable		*n*	Bypass	*n*	Sleeve	*p* value
Demographics (*n* = 347)			Mean (%)		Mean (%)	
Sex	Male	292	49 (16.8)	55	15 (27.3)	0.0793
	Female		243 (83.2)		40 (72.7)	
State	West Virginia	291	230 (79.0)	55	45 (81.8)	0.6314
	Others		61 (21.0)		10 (18.2)	
Race	Caucasian only	292	274 (93.8)	55	54 (98.2)	01923
	Others		18 (6.2)		1 (1.8)	
Education	High school or less	277	117 (42.2)	53	14 (26.4)	0.1723
	Some college or associates		92 (33.2)		22 (41.5)	
	Bachelors		44 (15.9)		12 (22.6)	
	Postgrad, masters, Ph.D., and law		24 (8.7)		5 (9.4)	
Marital	Single	277	48 (17.3)	54	8 (14.8)	0.7696
	Married		176 (63.5)		38 (70.3)	
	Divorced		37 (13.4)		5 (9.3)	
	Others		16 (5.8)		3 (2.6)	

Diagnosed baseline psychological comorbidities		*n*	%	*n*	%	*p*

	ICD anxiety disorders	93	42.7	14	36.8	0.5216
	ICD depression	136	55.7	30	63.8	0.3048

Baseline measures		*n*	Mean (SD)	*n*	Mean (SD)	*p*

	Age (year)	292	44.9 (11.5)	55	45.3 (11.6)	0.8352
	Height (cm)	292	166.3 (8.9)	55	168.7 (9.7)	0.0445^*∗*^
	Weight (kg)	292	136.7 (27.3)	55	137.6 (26.3)	0.8548
	BMI (kg/m^2^)	292	49.0 (8.1)	55	48.0 (7.2)	0.3846
	EBW (kg)	292	67.0 (23.2)	55	66.6 (22.8)	0.7497
	Beck anxiety inventory (0–63)	176	6.3 (6.9)	45	6.2 (6.7)	0.8411
	Beck depression inventory (0–63)	205	11.0 (9.6)	47	10.7 (7.2)	0.5769

Six-month measures		*n*	Mean (SD)	*n*	Mean (SD)	*p*

	Weight (kg)	185	98.7 (22.0)	39	106.0 (23.6)	0.0143^*∗*^
	%TWL	185	28.1 (6.2)	39	21.6 (6.2)	<0.0001^*∗*^
	BMI (kg/m^2^)	185	35.9 (6.2)	39	38.1 (6.8)	0.0509
	ΔBMI	185	13.7 (14.2)	39	10.0 (3.8)	<0.0001^*∗*^
	EBW (kg)	185	29.6 (18.9)	39	36.4 (19.9)	0.0383^*∗*^
	%EWL	185	59.6 (15.6)	39	46.9 (15.7)	<0.0001^*∗*^

Year-one measures		*n*	Mean (SD)	*n*	Mean (SD)	*p*

	Weight (kg)	160	89.9 (20.0)	24	101.9 (18.9)	0.0016^*∗*^
	%TWL	160	34.5 (7.7)	24	24.0 (6.6)	<0.0001^*∗*^
	BMI (kg/m^2^)	160	32.9 (6.1)	24	36.4 (6.1)	0.0095^*∗*^
	ΔBMI	160	16.9 (5.9)	24	11.3 (4.4)	<0.0001^*∗*^
	EBW (kg)	160	21.1 (17.1)	24	31.8 (16.6)	0.0028^*∗*^
	%EWL	160	72.2 (17.9)	24	51.7 (15.2)	<0.0001^*∗*^

Two-year measures		*n*	Mean (SD)	*n*	Mean (SD)	*p*

	Weight (kg)	97	91.6 (21.3)	12	111.1 (20.9)	0.0032^*∗*^
	%TWL	97	33.8 (9.8)	12	19.0 (10.2)	<0.0001^*∗*^
	BMI (kg/m^2^)	97	33.2 (6.4)	12	39.4 (7.5)	0.0062^*∗*^
	ΔBMI	97	17.3 (7.5)	12	7.0 (5.3)	<0.0001^*∗*^
	EBW (kg)	97	22.7 (18.3)	12	38.2 (18.5)	0.0061^*∗*^
	%EWL	97	69.8 (21.1)	12	41.2 (21.2)	0.0002^*∗*^

Chi-square analysis was used for assessing demographic variables with two groups by surgery type. Fisher's exact test was used for cell sizes <5. Mann–Whitney *U* test was used for examining the relationship of nonparametric continuous variables by surgery type (age, height, weight, %TWL, BMI, ΔBMI, EBW, %EWL, BAI, and BDI). ^*∗*^Significant at <0.05 level.

**Table 2:** Mixed regression models for percent of excess body weight loss.

Variable	Category	Estimate	SE	*t*-Value	*p* value
Model 1%EWL (*n* = 259)					
Intercept		48.17	6.00	8.03	<0.0001^*∗*^
Gender (referent: male)	Female	7.19	2.55	2.82	0.0052^*∗*^
Age		−0.15	0.09	−1.57	0.1174
Race (referent: white)	Others	2.00	4.45	0.45	0.6533
Surgery (referent: sleeve)	Bypass	7.11	3.91	1.82	0.0693
BDI-II		−0.08	0.12	−0.70	0.4839
Time		0.29	0.85	0.35	0.7300
Time*∗*surgery	Bypass	3.31	0.93	3.55	0.0004^*∗*^

Model 2%EWL (*n* = 228)					
Intercept		44.54	6.28	7.09	<0.0001^*∗*^
Gender (referent: male)	Female	8.48	2.78	3.16	0.0018^*∗*^
Age		−0.10	0.10	−0.97	0.3324
Race (referent: white)	Others	−0.41	4.82	−0.09	0.9322
Surgery (referent: sleeve)	Bypass	8.28	4.00	2.07	0.0394^*∗*^
BAI		−0.14	0.17	−0.83	0.4084
Time		0.53	0.84	0.63	0.5296
Time*∗*surgery	Bypass	3.33	0.94	3.53	0.0005^*∗*^

Linear mixed model adjusted for age, gender, and race/ethnicity was used to analyze the main effects of surgery (bypass and sleeve), time, and self-reported mood scale (BDI-II or BAI) and their interaction on %EWL. Significant effects were followed by multiple comparisons using Tukey adjustment. *p* values for the main effects and interaction are indicated. ^*∗*^*p* < 0.05.

## Data Availability

The data used to support the findings of this study are available from the corresponding author upon request.
